# Efficacy and safety of tacrolimus combined with low-dose glucocorticoids *vs.* high-dose glucocorticoids in initial treatment of adult-onset minimal change disease: a retrospective cohort study

**DOI:** 10.7717/peerj.21089

**Published:** 2026-04-13

**Authors:** Xiaotao Ma, Jiayi Li, Xia Yang, Baoling Li, Tian Yao, Lifang Tian, Fuqian Lei, Xiaoyong Yu, Shohida Naimova, Sui Lin Cao, Rongguo Fu

**Affiliations:** 1 Department of Nephrology, Second Affiliated Hospital of Xi’an Jiaotong University, Xi’an, Shaanxi, China; 2 Department of Nephrology, Shaanxi Traditional Chinese Medicine Hospital, Xi’an, Shaanxi, China; 3 Department of Nephrology, Bukhara State Medical Institute, Bukhara, Uzbekistan; 4 Department of Nephrology, Xi’an Gaoxin Hospital, Xi’an, Shaanxi, China

**Keywords:** Minimal change disease, Glucocorticoids, Tacrolimus, Nephrotic syndrome, Adult

## Abstract

**Background:**

The treatment of adult minimal change disease (MCD) is challenging due to the side effects of high-dose and long-term glucocorticoid therapy as well as the frequent relapsing of the disease. Clinically, there is a demand for regimens that ensure rapid response and lower relapse rates, such as calcineurin inhibitors could fulfill this role. This study aims to compare the efficacy and safety of tacrolimus combined with low-dose glucocorticoids *vs.* high-dose glucocorticoids in the treatment of newly diagnosed adult MCD.

**Methods:**

A retrospective cohort study was conducted on 59 adult patients diagnosed with MCD *via* renal biopsy at the Second Affiliated Hospital of Xi’an Jiaotong University, China. Patients were divided into two groups: the high-dose glucocorticoids group (GCs group, 39 patients, receiving 1 mg/kg/day prednisone) and the tacrolimus combined with low-dose glucocorticoid group (TAC group, 20 patients, receiving 0.05 mg/kg/day tacrolimus plus 10 mg/day prednisone). Both groups were followed for at least 24 weeks. The primary endpoint was the complete remission rate at 24 weeks, with secondary endpoints including relapse rates. And adverse events was analysed.

**Results:**

At 24 weeks, the complete remission rates (90% *vs*. 100%, *p* = 0.111) and cumulative relapse rates (5% *vs*. 25.6%, *p* = 0.054) were comparable between the TAC and GCs groups. Patients receiving high-dose glucocorticoids were more likely to show incurred higher Cushingoid features (41% *vs*. 5%, *p* = 0.004). Patients in the tacrolimus group had a higher risk of transient serum creatinine elevation (10% *vs*. 0%, *p* = 0.045), resolving spontaneously. Longitudinal albumin recovery and estimated glomerular filtration rate (eGFR) stability were similar between groups.

**Conclusions:**

Tacrolimus combined with low-dose glucocorticoids demonstrates comparable efficacy to high-dose glucocorticoids in inducing remission for adult-onset MCD, with a more favorable safety profile.

## Introduction

Minimal change disease (MCD), a leading cause of adult-onset nephrotic syndrome, accounts for 10–25% of cases globally (Vivarelli et al., 2017), with a lower prevalence reported in China (13%) (Hu et al., 2020; Wang et al., 2024). Its pathogenesis involves immune dysregulation, characterized by interleukin-13 overexpression, regulatory T-cell dysfunction, and autoantibodies targeting antigens on podocyte antigens (*e.g*., nephrin and annexin A2) (Campbell & Thurman, 2022; Eddy & Symons, 2003; Hengel et al., 2024; Watts et al., 2022; Ye et al., 2021). Clinically, MCD is associated with complications such as acute kidney injury (AKI), thromboembolism, and hyperlipidemia, which collectively contribute to morbidity and hospitalization risks (Meyrier & Niaudet, 2018; Vivarelli et al., 2017). The Kidney Disease: Improving Global Outcomes (KDIGO) guidelines endorse high-dose glucocorticoids (1 mg/kg/day) as first-line therapy for adult MCD, despite limited evidence supporting this recommendation (Grade 1C) (Rovin et al., 2021). While effective, prolonged steroid use in adults is complicated by high relapse rates (56–76%) (Nolasco et al., 1986; Waldman et al., 2007) and substantial toxicity, including Cushing’s syndrome, metabolic disturbances, and opportunistic infections. These challenges necessitate steroid-sparing strategies, particularly for patients with comorbidities like diabetes or osteoporosis (Huscher et al., 2009; Oray et al., 2016).

Current research predominantly focuses on frequently relapsing or steroid-dependent (FR/SD) MCD, where calcineurin inhibitors (CNIs), mycophenolate mofetil (MMF), and cyclophosphamide (CTX) are used as second-line agents (Meyrier, 1989). Among CNIs, tacrolimus (TAC) has emerged as a preferred option due to its dual mechanism: suppressing T-cell activation and stabilizing podocyte cytoskeletal integrity to reduce proteinuria (Faul et al., 2008). Compared to cyclosporine A (CsA), tacrolimus demonstrates comparable efficacy with lower risks of cosmetic side effects and hyperlipidemia (Fan et al., 2013; Shah & Hafeez, 2016). However, evidence for TAC in *de novo* MCD is limited to small cohorts or retrospective designs (Li et al., 2017; Xu et al., 2017). Preliminary data suggest that tacrolimus monotherapy achieves remission rates comparable to glucocorticoids, with fewer adverse events. Li et al. (2017) reported a remission rate of 92.5%, slightly higher than the 85% reported by Medjeral-Thomas et al. (2020). In contrast, a randomized controlled trial conducted by Chin et al. (2021) demonstrated that the remission rate in the tacrolimus combined with low-dose glucocorticoids group was significantly higher than that in the standard glucocorticoid group (93.8% *vs*. 73.3%, respectively), and also higher than the remission rates observed in the aforementioned tacrolimus monotherapy studies. These findings suggest a synergistic effect between tacrolimus and glucocorticoids.

Here, we conducted a retrospective study comparing tacrolimus combined with low-dose prednisone (10 mg/day) *vs*. high-dose glucocorticoids (1 mg/kg/day) in adults with newly diagnosed MCD, aiming to evaluate efficacy, relapse rates, and safety profiles.

## Materials and Methods

### Patients

All patients were followed at the Second Affiliated Hospital of Xi’an Jiaotong University. The study was approved by the Institutional Review Board of the Second Affiliated Hospital of Xi’an Jiaotong University (Approval No. 2025 Lun Shen 075). As a retrospective analysis of existing clinical data, this study was not registered in a public trial registry. Before participation in this study, written informed consent was obtained from all patients and their parents/guardians.

### Inclusion and exclusion criteria

Inclusion criteria comprised an age of over 18 years, a diagnosis of nephrotic syndrome with MCD, and a follow-up period of more than 24 weeks. Exclusion criteria included the presence of infections such as hepatitis B, hepatitis C, HIV, syphilis, and other untreated infections. Pregnant or breastfeeding women, those at risk of pregnancy, and patients with secondary causes of nephrotic syndrome were also excluded.

### Group assignment

The group assignment was based on the initial treatment decision made by the doctors in real-world clinical practice, not by random allocation. The decision was primarily guided by the following clinical considerations: (1) patient preference and concern about potential side effects of high-dose glucocorticoids; (2) the presence of relative contraindications or high-risk factors for glucocorticoids (*e.g*., poorly controlled diabetes, obesity, psychiatric history, or prior intolerance); and (3) the comprehensive judgment of the clinician integrating the patient’s overall condition and the treatment patterns at our center. At our center during the study period, both treatment regimens (the high-dose glucocorticoids and the tacrolimus combined with low-dose glucocorticoid) were considered standard first-line or alternative first-line options for adult MCD.

Based on their initial treatment, patients were divided into two groups: the high-dose glucocorticoids group (GCs group, 1 mg/kg) and the tacrolimus combined with low-dose glucocorticoid group (TAC group, 10 mg/d). The treatment regimen for the GCs group started with an initial dose of prednisone (1 mg/kg), with a maximum dose not exceeding 70 mg/day. Prednisone was reduced by 5 mg per week starting 1 week after achieving complete remission, with the aim to discontinue within 6 months, but at least within 16 weeks. During treatment, patients received oral omeprazole (20 mg twice daily) and calcium carbonate (600 mg/tablet, two tablets daily). In the TAC group, the initial dose of tacrolimus was 0.05 mg/kg twice daily combined with prednisone (10 mg/d). The target trough blood level of tacrolimus was maintained at 5–10 ng/ml. Tacrolimus was tapered and discontinued within 6 months, starting 2 weeks after complete remission. Both groups received basic treatment for hypertension with angiotensin receptor blockers or angiotensin-converting enzyme inhibitors, and treatment for hypercholesterolemia with statins. To prevent thromboembolic events, low molecular weight heparin was administered subcutaneously if albumin levels were less than 20 g/L, and aspirin (75 mg daily) was taken orally when albumin levels were between 20 and 30 g/L. During the treatment period, sulfamethoxazole dispersive was used to prevent *Pneumocystis carinii* infection in case of hypoimmunity.

### Definitions

Nephrotic syndrome was defined as plasma albumin levels below 30 g/L and 24-h urinary protein excretion exceeding 3.5 g, with or without hyperlipidemia. Complete response (CR) was defined as urinary protein excretion less than 500 mg within 24 h. Relapse (RP) was defined as an increase in urinary protein excretion above 3,500 mg after complete remission or return to nephrotic syndrome. The relapse rate at any given time point was defined as the proportion of patients within a group meeting the relapse criteria at that time. Acute kidney injury (AKI) is defined as an increase in serum creatinine (Scr) of ≥0.3 mg/dL within 48 h or a 1.5-fold increase in Scr within 7 days from the baseline value or a urine output of <0.5 mL/(kg·h) for at least 6 h. The primary outcome was complete remission of nephrotic syndrome at 24 weeks. Secondary outcome were rates of relapse of nephrotic syndrome.

### Clinical and biological data

Follow-up records of demographic characteristics and laboratory tests. We conducted a comprehensive review and collected clinical, biological and histopathological at the initiation of treatment and the every-month follow-ups to assess complete remission and relapse. These data were obtained from the reports of the clinical laboratory of the Second Affiliated Hospital of Xi’an Jiaotong University.

### Statistical analysis

SPSS 26 (IBM Corp, Armonk, NY, USA) was used for statistical analysis. Continuous data conforming to a normal distribution were expressed as mean ± standard deviation and compared using t-test or one-way ANOVA. For continuous data with skewed distributions, the Mann-Whitney U test was employed. Categorical data were analyzed using chi-square tests and Fisher’s exact tests. Inter-group comparisons were adjusted for multiple comparisons using the Bonferroni correction. Survival analysis was conducted using the Kaplan-Meier estimation method. A *p*-value of less than 0.05 was considered statistically significant.

## Results

### Baseline data

**Figure 1:**
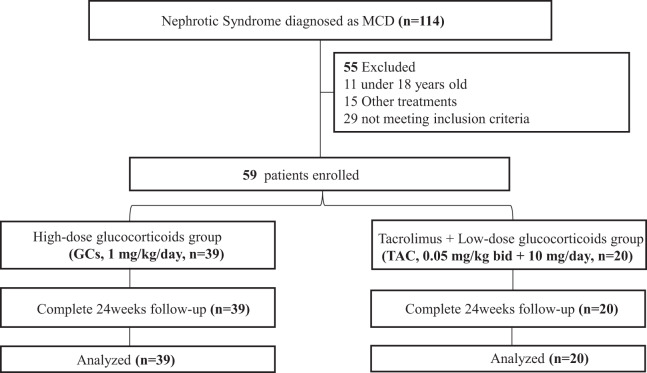
Inclusion and grouping of MCD in adults.

A total of 114 patients with biopsy-confirmed MCD were initially screened at our institution between June 2018 and June 2024. After applying inclusion and exclusion criteria, 59 patients were enrolled. They were grouped according to the initial clinical treatment strategy, and 39 allocated to the GCs group and 20 to the TAC group. The detailed patient selection process is depicted in Fig. 1.

Baseline demographic and clinical characteristics were well-balanced between the two cohorts (Table 1). The overall study population had a median age of 41 years (range: 18–83 years), with a slight male predominance (56.24% males *vs*. 43.76% females). No significant differences were observed in sex distribution (GCs group: 56.41% males *vs*. TAC group: 45% males, *p* = 0.406), age (median: 42 *vs*. 41 years, *p* = 0.471), or blood pressure (systolic: 120.28 ± 14.55 *vs*. 119.05 ± 14.95 mmHg, *p* = 0.762; diastolic: 77.97 ± 10.70 *vs*. 80.80 ± 11.23 mmHg, *p* = 0.349).

**Table 1:** Comparison of baseline data between the GCs group and TAC group.

Variable	Total (*n* = 59)	GCs group (*n* = 39)	TAC group (*n* = 20)	Test value	*P* value
Male, *n* (%)	32 (54.24%)	22 (56.41%)	9 (45.0%)	0.690	0.406
Age (years)	41 (26, 56)	42 (23, 56)	41 (30, 59.75)	−0.721	0.471
SBP (mmHg)	119.86 ± 14.58	120.28 ± 14.55	119.05 ± 14.95	0.305	0.762
DBP (mmHg)	78.93 ± 10.88	77.97 ± 10.70	80.80 ± 11.23	−0.944	0.349
HB (g/L)	142.25 ± 17.69	140.92 ± 17.12	144.85 ± 18.91	−0.805	0.424
UTP (g/24 h)	7.88 (5.76, 10.11)	7.60 (5.76, 9.69)	8.13 (5.25, 11.52)	−0.624	0.532
ALB (g/L)	20.57 ± 4.37	20.19 ± 4.17	21.31 ± 4.77	−0.926	0.358
Scr (μmol/L)	60.55 (54.29, 72.48)	61.30 (54.10, 72.75)	58.18 (55.04, 69.59)	−0.352	0.725
eGFR (ml/min/1.73 m^2^)	109.94 (95.43, 131.89)	106.77 (95.43, 128.8)	113.78 (93.68, 132.23)	−0.544	0.586
TC (mmol/L)	9.63 ± 3.17	9.88 ± 3.23	9.12 ± 3.05	0.872	0.387
TG (mmol/L)	2.14 (1.42, 2.83)	2.09 (1.40, 3.0)	2.33 (1.53, 2.83)	−0.496	0.620
BUA (μmol/L)	372.00 (286.0, 462)	347.00 (277, 476)	391.50 (302.75, 444.50)	−0.593	0.554
IgG (g/L)	5.75 (3.64, 6.80)	5.48 (3.50, 7.04)	5.78 (3.82, 6.76)	−0.400	0.689

**Note:**

Abbreviations: SBP, Systolic Blood Pressure; DBP, Diastolic Blood Pressure; HB, Hemoglobin; UTP, Urine Total Protein; Scr, Serum creatinine; eGFR, estimate glomerular filtration rate calculated by CKD-EPI; TC, Total Cholesterol; TG, Triglycerides; BUA, Blood Uric Acid; ALB, Albumin; IgG, Immunoglobulin G.

Laboratory parameters at baseline revealed subtle intergroup differences. The GCs group exhibited lower median urinary total protein (7.60 g/24 h *vs*. 8.13 g/24 h, *p* = 0.532) and serum albumin levels (20.19 ± 4.17 g/L *vs*. 21.31 ± 4.77 g/L, *p* = 0.358) compared to the TAC group. Conversely, serum creatinine levels were marginally higher in the GCs group (61.30 μmol/L *vs*. 58.18 μmol/L, *p* = 0.725), accompanied by a reduced estimated glomerular filtration rate (eGFR: 106.77 *vs*. 113.78 mL/min, *p* = 0.586), likely attributable to a higher incidence of acute kidney injury (AKI) in this cohort (6 *vs*. 3 cases). To investigate the impact of serum creatinine levels and eGFR on MCD relapse, binary logistic regression was employed, which revealed no significant association (*p* > 0.05). Blood lipid profiles, including total cholesterol (9.88 ± 3.23 *vs*. 9.12 ± 3.05 mmol/L, *p* = 0.387) and triglycerides (median: 2.09 *vs*. 2.33 mmol/L, *p* = 0.620), as well as immunoglobulin levels (IgG: 5.48 *vs*. 5.78 g/L, *p* = 0.689), showed no statistically significant differences.

Notably, despite these baseline variations, all parameters remained within clinically comparable ranges, supporting the homogeneity of the study population. The observed disparities in eGFR and albumin levels may reflect the transient renal complications in the GCs group rather than intrinsic group imbalances.

### Comparison of the rates of complete remission between the two groups

**Figure 2:**
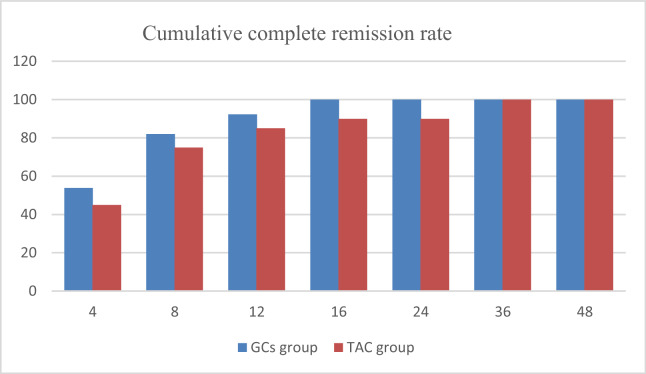
Cumulative complete remission rate of two groups.

At 24 weeks, the GCs group achieved a CR rate of 100% compared to 90% in the TAC group (Fig. 2), though this difference did not reach statistical significance (*p* = 0.111). Early response rates also favored the GCs group, with CR rates of 30.8% *vs*. 20% at 4 weeks and 82.1% *vs*. 75% at 8 weeks, respectively; however, these differences similarly lacked statistical significance (both *p* > 0.05). Notably, the median time to CR was markedly shorter in the GCs group (7 days *vs*. 14 days), with the earliest remission occurring within 3 days.

### Comparison of the rates of relapse between the two groups

**Figure 3:**
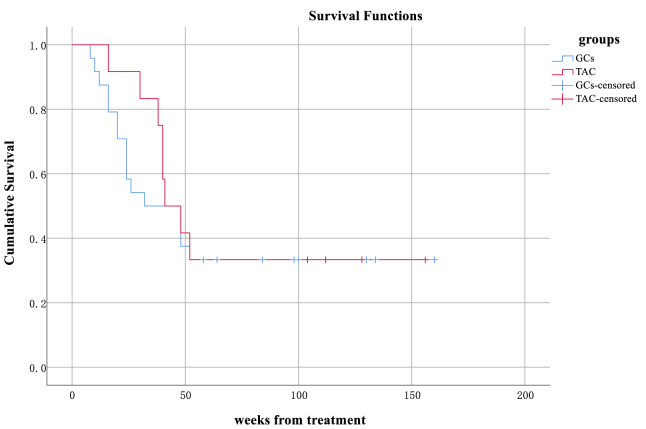
Survival analysis of adults with MCD treated in the GCs and TAC groups.

Longitudinal follow-up highlighted divergent relapse patterns. By 24 weeks, the cumulative relapse rate in the GCs group was 25.6% (10/39), compared to only 5% (1/20) in the TAC group (*p* = 0.054), a difference approaching statistical significance (Fig. 3). Analysis by Fisher’s exact test yielded no significant intergroup differences in the 24-week relapse rates (*p* = 0.079, OR = 0.153, 95% CI [0.018–1.291]). Although the Kaplan–Meier curve suggested a numerically higher relapse rate in the GCs group than in the TAC group, the log-rank test showed no statistically significant difference (χ^2^ = 0.218, df = 1, *p* = 0.64). Therefore, based on the present data, high-dose glucocorticoids were not associated with an increased relapse rate.

Over the entire observation period, relapse events in the GCs group clustered within three intervals: 10 cases within 24 weeks, six cases between 24 and 52 weeks, and nine cases beyond 52 weeks. In contrast, the TAC group exhibited fewer early relapses (one case within 24 weeks) but a gradual increase thereafter (seven cases at 24–52 weeks and four cases after 52 weeks). These trends align with prior findings by Medjeral-Thomas et al. (2020).

### Changes of plasma albumin, 24h urinary protein and eGFR during follow-up

**Figure 4:**
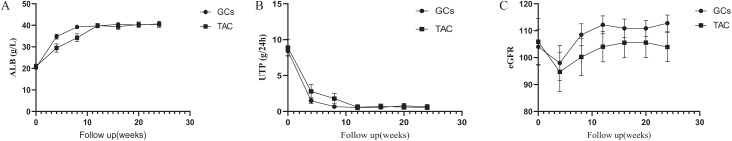
Changes in albumin, 24-h urinary protein, and eGFR during treatment in adults with MCD in the GCs and TAC groups. (A) Changes in serum albumin levels (ALB). (B) Changes in 24-h urinary protein quantification (UTP). (C) Changes in eGFR.

During the follow-up period, no significant differences were observed in plasma albumin (ALB), eGFR, and 24-h urinary protein levels (UTP) between the GCs group and the TAC group. Specifically, at 4 weeks and 8 weeks, the ALB levels were higher in the GCs group compared to the TAC group (Fig. 4A). After Bonferroni multiple comparison correction, the inter-group differences at all time points did not reach the significance level.

In terms of 24-h urinary protein levels, the TAC group exhibited higher levels compared to the GCs group before 12 weeks, though the differences were not statistically significant. At the 4 and 8 weeks, the 24-h urinary protein levels in the GCs group were 1.49 ± 0.4 g and 0.67 ± 0.23 g, respectively. In contrast, the corresponding levels in the TAC group were higher, 2.79 ± 0.95 g and 1.79 ± 0.72 g, respectively. Beyond the 12-week mark, the levels of 24-h urinary protein equalized between the two groups (Fig. 4B).

Although eGFR was lower in the TAC group compared to the GCs group during the treatment period, both groups maintained relatively stable average eGFR levels throughout the follow-up (Fig. 4C). The initial lower eGFR observed in the GCs group was attributed to six patients who experienced acute kidney failure and three patients who underwent kidney replacement therapy. Notably, all patients with acute kidney failure achieved normal kidney function following treatment.

These findings suggest that while there may be early differences in ALB levels between the treatment groups, long-term kidney function and proteinuria outcomes are comparable between glucocorticoid and tacrolimus treatments. This supports the potential for both treatment strategies to stabilize key kidney parameters in patients with minimal change disease during follow-up.

### Comparison of safety and adverse events between the two groups

The safety profiles of the two regimens diverged markedly (Table 2). Cushingoid features, including moon faces, were significantly more prevalent in the GCs group (41.0% [16/39] *vs*. 5.0% [1/20], *p* = 0.004), consistent with the metabolic consequences of prolonged high-dose glucocorticoid exposure. Conversely, transient elevations in serum creatinine occurred exclusively in the TAC group (10.0% [2/20] *vs*. 0%, *p* = 0.045), though all cases resolved spontaneously without clinical intervention.

**Table 2:** Adverse events in adults with MCD in the GCs and TAC groups.

Adverse event	GCs group (*n* = 39)	TAC group (*n* = 20)	$\chi^{2}$ value	*P* value
Cushingoid features	41.0% (16/39)	5.0% (1/20)	8.365	0.004
Buffalo hump	10.3% (4/39)	0% (0/20)	2.200	0.138
Upper respiratory infection	7.7% (3/39)	10.0% (2/20)	0.091	0.763
Herpes zoster	5.1% (2/39)	5.0% (1/20)	<0.001	0.983
New-onset diabetes	10.3% (4/39)	10.0% (2/20)	0.001	0.975
Transient kidney dysfunction	0% (0/39)	10.0% (2/20)	4.037	0.045
Static tremor	7.7% (3/39)	20.0% (4/20)	1.915	0.166
Osteoporosis	5.1% (2/39)	5.0% (1/20)	<0.001	0.983

Other adverse events showed no statistically significant intergroup differences. Infections, including upper respiratory tract infections (7.7% [3/39] *vs*. 10.0% [2/20], *p* = 0.763) and herpes zoster (5.1% [2/39] *vs*. 5.0% [1/20], *p* = 0.983), occurred at comparable rates. Metabolic complications such as new-onset diabetes (10.3% [4/39] *vs*. 10.0% [2/20], *p* = 0.975) and musculoskeletal changes (*e.g*., buffalo hump: 10.3% [4/39] *vs*. 0%, *p* = 0.138) were infrequent and did not differ between groups. Neurological side effects, including static tremors (7.7% [3/39] *vs*. 20.0% [4/20], *p* = 0.166), and osteoporosis (5.1% [2/39] *vs*. 5.0% [1/20], *p* = 0.983) were similarly balanced between the GCs and TAC groups.

## Discussion

Recent studies have shown that MCD accounts for up to 15% of adult nephrotic syndrome cases (Hu et al., 2020; Vivarelli et al., 2017; Wang et al., 2024). Unlike in children, adults with MCD often exhibit lower rates of complete remission and shorter durations of remission (Chen et al., 2023). Consequently, adults frequently receive higher cumulative doses of corticosteroids, which increases the risk of associated adverse effects. This has driven nephrologists to explore new treatments that are both more effective and have fewer side effects. While most studies have focused on FR/SD-MCD in children, improving the complete remission rate and reducing the relapse rate of initial MCD treatment in adults offers significant clinical, economic, and social benefits.

Current initial treatment options for adults with MCD include glucocorticoids (administered orally or intravenously, daily or on alternate days), tacrolimus (used alone or in combination with glucocorticoids) and anti-CD20 monoclonal antibodies. Although many recent studies have shown that anti-CD20 antibodies can also rapidly alleviate adult MCD and reduce the recurrence rate, for patients with poor economic conditions and those who have difficulty accessing the monoclonal antibodies, they still mainly rely on non-biological drugs. Our retrospective study may be helpful to them.

Maruyama et al. (1994) demonstrated that tacrolimus can reduce the production of vascular permeability factors in the serum of patients with MCD, providing scientific evidence for its use in MCD treatment. In 1997, Schweda et al. (1997) was the first to report that tacrolimus successfully treated MCD patients with steroid- and cyclosporin-resistant nephrotic syndrome. To further evaluate whether tacrolimus is more effective and safer than CsA in inducing remission in patients with steroid-resistant nephrotic syndrome (SRNS), a randomized controlled trial was conducted. The trial found similar remission rates at 6 months (85.7% *vs*. 80%) and 12 months between tacrolimus and CsA, but significantly fewer relapses among those receiving tacrolimus. Cosmetic side effects were more frequent in CsA-treated patients (Choudhry et al., 2009). A prospective multicenter trial demonstrated that tacrolimus combined with prednisone can serve as an effective alternative regimen for adult SRNS patients in China, achieving a 58.3% complete remission rate and a 16.7% partial remission rate after 6 months of treatment—even in cases beyond minimal change disease (Fan et al., 2013).

What about the role of tacrolimus in adults with newly diagnosed MCD? In a randomized study of adult-onset minimal change nephrotic syndrome in China, patients received glucocorticoid or tacrolimus after intravenous methylprednisolone. Both groups showed similar total remission rates (96.2% *vs*. 98.3%) (Li et al., 2017). This suggests that remission rate is high when tacrolimus monotherapy after short-term intravenous methylprednisolone. In order to investigate whether tacrolimus monotherapy without corticosteroids would be effective for the treatment of *de novo* minimal change disease. A UK multi-center RCT also compared tacrolimus with prednisolone for the treatment of patients with nephrotic syndrome and found no significant difference in the rate of complete remission between the two groups (Medjeral-Thomas et al., 2020). Our complete remission rate is higher than this group at 8 weeks (75% *vs*. 68%), 16 weeks (90% *vs*. 76%), 24 weeks (90% *vs*. 88%) in the TAC group. And the complete remission rates of the two prednisolone groups were similar. This indicates that the combination of tacrolimus and prednisone (10 mg daily) can promote remission. Chin et al. (2021) explored tacrolimus combined with low-dose steroids (0.5 mg/kg daily) for adult MCD showed comparable complete remission rates within 8 weeks between the combination group (79.1%) and the high-dose steroid group (76.8%). Comparative analysis indicated that despite using a lower steroid dose (10 mg/d prednisone) in our TAC group, the 8-week complete remission rate (75% *vs*. 79.1%) and cumulative relapse rate (5% *vs*. 5.7%) remained comparable to those reported by Chin et al. (2021), suggesting low-dose steroid combined with tacrolimus is effective for MCD remission. Therefore, our data proved that even 10 mg/d of prednisone can work in conjunction with tacrolimus to achieve remission of MCD.

The time to complete remission was shorter in the GCs group (4 weeks) than the TAC group (5.5 weeks). One study found that the response time was shorter in children with MCD treated with methylprednisolone (Imbasciati et al., 1985). Similarly, Wang et al.’s (2025) retrospective analysis showed that the median response time to steroid therapy was 5 weeks. The more rapid remission observed in the corticosteroid group compared to the tacrolimus group may be attributed to the direct action of corticosteroids on cytokines and T cells, whereas tacrolimus requires time to achieve therapeutic blood concentrations and stabilize the cytoskeleton.

The side effects of glucocorticoids and tacrolimus have been well-documented in previous studies. In our study, glucocorticoids were more likely to cause facial changes, like moon-shaped faces, while tacrolimus was associated with transient nephrotoxicity. There was little difference in infectious events between the two groups.

However, this study has some limitations. First, the study is limited by its retrospective, non-randomized, open-label design and the small sample size, which may increase the risk of type II error and could explain the absence of a significant difference in relapse rates. Although selection bias is inherent in such a design, the baseline characteristics were well balanced between the two groups (Table 1), suggesting that its potential impact on the statistical outcomes is likely limited. Furthermore, a Bonferroni correction was applied to adjust for multiple comparisons. It should also be noted that sample sizes in comparable studies in this field typically range from 20 to 30 patients (Choudhry et al., 2009; Fan et al., 2013; Medjeral-Thomas et al., 2020). Second, the interpretability of the findings is limited by the lack of detailed records on several parameters: tacrolimus trough levels (which were not consistently monitored), timing of tacrolimus withdrawal, individual prednisone tapering speed, and autoantibody assessments. Third, the follow-up period was relatively short, although it was selected based on endpoints commonly used in prior studies (Li et al., 2017; Medjeral-Thomas et al., 2020). We plan to conduct a prospective randomized controlled study with at least 52 weeks of follow-up to address these limitations.

## Conclusions

In summary, our study suggests that tacrolimus combined with low-dose glucocorticoids (10 mg/d) has similar efficacy to high-dose glucocorticoids (1 mg/kg) in treating adults with initial MCD while offering fewer adverse effects. Treatment choice for adult MCD should consider underlying diseases and renal function to optimize patient outcomes.

## Supplemental Information

10.7717/peerj.21089/supp-1 Supplemental Information 1 Raw data for baseline. Each row represents a patient’s baseline data before treatment.

10.7717/peerj.21089/supp-2 Supplemental Information 2 Raw data for follow up. Follow-up data for ALB, UTP, and eGFR from patients in the GCs and TAC groups.

10.7717/peerj.21089/supp-3 Supplemental Information 3 Raw data for replase and complete remission. Cumulative relapse and complete remission in patients from the GCs and TAC groups.

10.7717/peerj.21089/supp-4 Supplemental Information 4 Codebook.

## Additional Information and Declarations

### Data Availability

The following information was supplied regarding data availability:

The raw data are available in the Supplemental Files.

### Clinical Trial Registration

The following information was supplied regarding Clinical Trial registration:

As a retrospective analysis of existing clinical data, this study was not registered in a public trial registry.
