# Supplementary material for: Efficacy and safety of tacrolimus combined with low-dose glucocorticoids vs. high-dose glucocorticoids in initial treatment of adult-onset minimal change disease: a retrospective cohort study
Source: PeerJ. 2026 Apr 13;14:e21089. doi: 10.7717/peerj.21089 (PMC13086028; doi:10.7717/peerj.21089)
Supplement: Supplemental Information 4 [file peerj-14-21089-s004.pdf]

This codebook describes the meaning of numerical codes used for categorical variables in the raw data file.

**Variable**	Meaning of Numerical Code
group	0=GCs group, 1=TAC group
replase	0=No, 1=Yes
gender	1=male, 2=female

The following list denotes the variable abbreviations and their corresponding full names in the raw data file.

Abbreviation	Full name
SBP(mmHg)	Systolic Blood Pressure
DBP(mmHg)	Diastolic Blood Pressure
HB(g/L)	Hemoglobin
UTP (g/24h)	Urine Total Protein
Scr(μmol/L)	Serum creatinine
eGFR(ml/min/1.73m^2)	Estimated Glomerular Filtration Rate
TC(mmol/L)	Total Cholesterol
TG(mmol/L)	Triglycerides
BUA(μmol/L)	Blood Uric Acid
ALB(g/L)	Albumin
IgG (g/L)	Immunoglobulin G
CR	Complete remission
NA	Not Applicable
